# Cholera Fungus

**Published:** 1868-10

**Authors:** 


Cholera Fungus.—We understand that the Director-General of the Army Medical Department and the Senate of the Army Medical School have taken an important step with a view to the final settlement of the Cholera Fungus question. Acting on the advice of the professors of the school, the authorities have consented to send two of the most distinguished élèves to Germany to study the subject under Professors Hallier and DeBary. The young medical officers, after mastering the mode of investigation pursued in this difficult inquiry by the eminent men above named, are to proceed to India, and to be set apart to investigate it in that great field of observation—in the very house of cholera. It is to be hoped that this well-advised measure will meet with the success it deserves. It is much to the credit of the Secretaries of State for War and India that they have consented to carry out this measure in a wise and liberal spirit.—*Lancet.*

